# Synthesis of 2D transition metal dichalcogenides by chemical vapor deposition with controlled layer number and morphology

**DOI:** 10.1186/s40580-018-0158-x

**Published:** 2018-09-28

**Authors:** Jiawen You, Md Delowar Hossain, Zhengtang Luo

**Affiliations:** Department of Chemical and Biological Engineering, Hong Kong University of Science and Technology, Clear Water Bay, Kowloon, Hong Kong

**Keywords:** Transition metal dichalcogenides (TMDs), 2D materials, Growth mechanisms, Chemical vapor deposition, Morphology

## Abstract

Two-dimensional (2D) transition metal dichalcogenides (TMDs) have stimulated the modern technology due to their unique and tunable electronic, optical, and chemical properties. Therefore, it is very important to study the control parameters for material preparation to achieve high quality thin films for modern electronics, as the performance of TMDs-based device largely depends on their layer number, grain size, orientation, and morphology. Among the synthesis methods, chemical vapor deposition (CVD) is an excellent technique, vastly used to grow controlled layer of 2D materials in recent years. In this review, we discuss the different growth routes and mechanisms to synthesize high quality large size TMDs using CVD method. We highlight the recent advances in the controlled growth of mono- and few-layer TMDs materials by varying different growth parameters. Finally, different strategies to control the grain size, boundaries, orientation, morphology and their application for various field of are also thoroughly discussed.

## Introduction

In the last decade, after tremendous success of the first two-dimensional (2D) material, i.e. graphene, transition metal dichalcogenides (TMDs) have attracted significant research attention due to their extraordinary properties. The TMDs represent a large family of layered materials with a generalize single layer formula as MX_2_, where M represents a transition metal from group IVB to group VIII (layered structures are usually found in group IVB to VII B, like Ti, V, Nb, Mo, W, Re), and X represents the chalcogen (S, Se, and Te) [1]. Compared with graphene, bulk TMDs show much wide range of physical properties, starting from insulators (e.g., HfS_2_) [2], semiconductors (e.g., MoS_2_, WS_2_) [3, 4], and semi metallics (e.g., WTe_2_ and TiSe_2_) [5, 6] to metallics (e.g., NbS_2_, VSe_2_) [7, 8]. Apart from this, a few of them (e.g., NbSe_2_, TaS_2_) shows unusual interesting properties like superconductivity, charge density wave (CDW, periodic crystal lattice distortion) and Mott transition (transition from metal to non-metal) [9–11]. However, mono- or few-layer TMDs exhibits other additional exciting electronics characteristics alone with abovementioned properties. As for example, monolayer MoS_2_ shows strong photoluminescence (PL) over bulk due to quantum confinements, confirmed by the change of bandgap from indirect to direct for bulk to monolayer MoS_2_ [12]. Further research also indicates that the CDW transition temperature of NbSe_2_ increases on reducing the layer number due to the enhanced electron–phonon interactions in 2D materials [13]. Due to numerous unique properties of thin layer TMDs materials, it has been used to fabricate new generation electronic [14–16] and optoelectronic devices [17–20] such as transistors, photodetector, photodiode, photovoltaic devices, etc. Therefore, researchers have devoted considerable efforts to synthesis high-quality and large-size TMDs thin layers, which plays a significant role in fundamental research and application exploration. Several techniques have been already introduced to synthesize atomically thin layer of high quality TMDs material such as mechanical exfoliation method [21, 22], liquid exfoliation method [23–25], chemical vapor deposition (CVD) method [26–29], wet chemical method [30, 31], etc. Among all synthesis techniques, the chemical vapor deposition (CVD) is promising for synthesis of high-quality TMDs layers with controllable layer number and domain size, as well as excellent properties, due to their simplicity and compatibility with industry standards.

In this review, we summarized some of the key and control factors affecting the growth of TMDs via CVD method. Here, CVD is used as a general term which covers the vapor transport and deposition methods. The growth mechanisms and synthesis approaches of mono- or few-layer TMDs are also be introduced. Moreover, we will thoroughly discuss the controllable growth of TMDs based on grain size/boundary, orientation, and morphology.

## Growth routes and mechanisms

Different strategies have been applied in the synthesis technique to obtain high quality large scale mono- and few-layer TMDs to explore their promising properties in various field of applications. Typically, all the synthesis methods have been classified into either “top-down” or “bottom-up” approaches [32]. Generally, exfoliation methods are followed “top-down” technique while CVD is based on “bottom-up” methods [33–35]. In a typical CVD process, precursors are reacted/or decomposed and deposited as mono- or few-layer film on the exposed substrate at relative high temperature. Many fundamental researches have been done to produce ultrathin TMDs materials with high crystal quality, scalable size, tunable thickness, and excellent electronic properties [26, 36, 37]. Compared with chemical vapor transport (CVT) method, which is commonly used to synthesize bulk single crystal materials, the typical CVD method we discussed in this review is for synthesizing mono- or few-layer TMDs. The reaction process is much quicker than CVT, and no subsequent mechanical exfoliation is required to obtain few layer structure for following study and application.

### Synthesis routes for TMDs layers

**Fig. 1:**
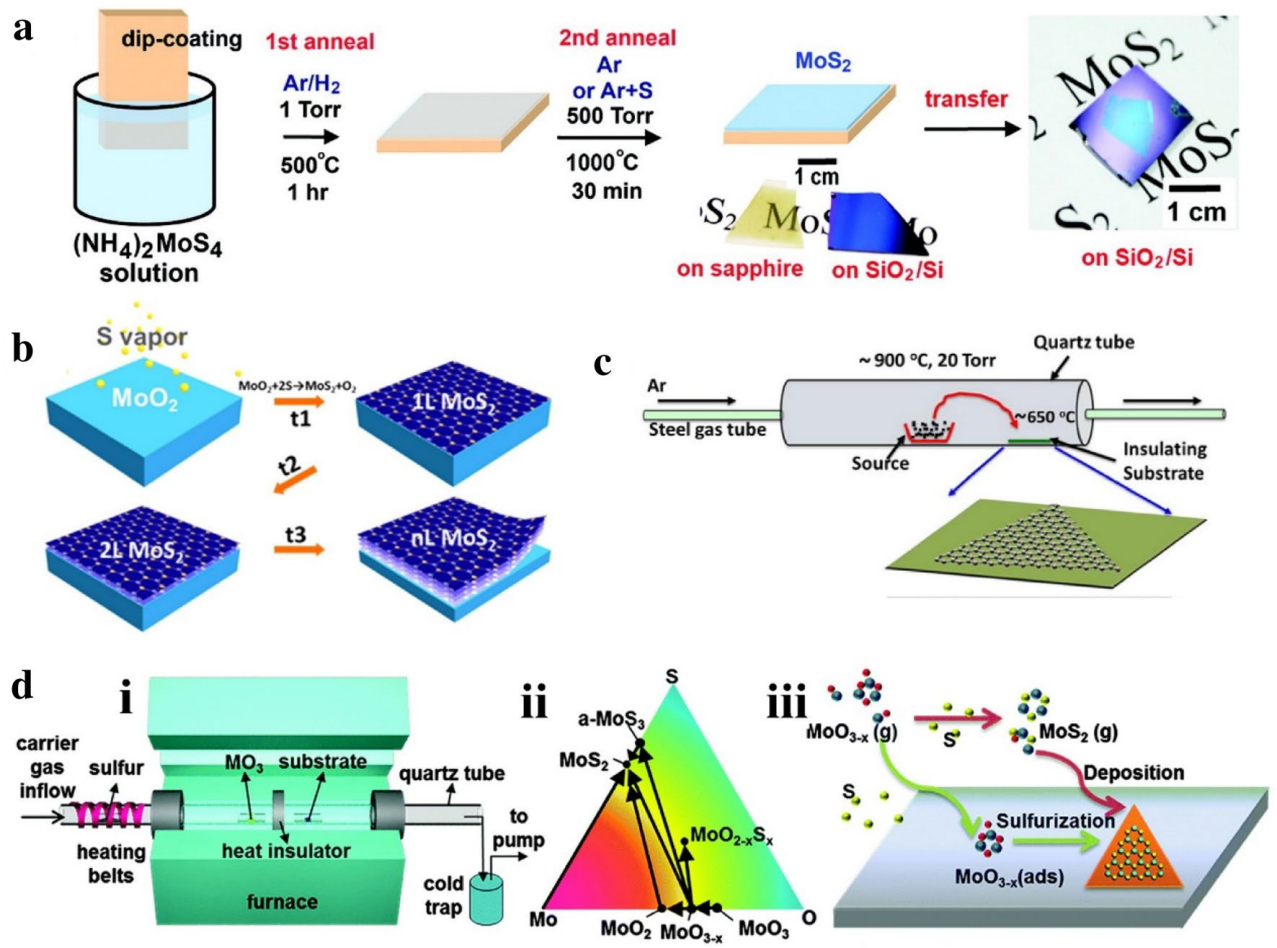
4 types of growth routes for TMDs. **a** Two-step thermolysis process synthesis from (NH_4_)_2_MoS_4_ precursor on SiO_2_/Si or sapphire substrates followed by the two-step annealing process on insulating substrates and finally transferred onto another substrate [39], Copyright 2012, American Chemical Society. **b** Layer by layer deposition of MoS_2_ from MoO_3_ by surface sulfurization and transferred to another substrate one by one [45], Copyright 2013, American Chemical Society. **c** Simple vapor transport method showing MoS_2_ films were deposited on insulating substrate at high temperature from MoS_2_ powder precursor under inert atmosphere [47], Copyright 2013, American Chemical Society. **d** (i) LPCVD system setup for TMDs, (ii) Mo–O–S ternary phase diagram illustrating the pathways for the CVD growth of MoS_2_ from MoO_3_ precursors, and (iii) Possible growth routes of MoS_2_ by the reaction of MoO_3−*x*_ and S [36]. Copyright 2015, Royal Society of Chemistry

There are four common routes of TMDs growth by CVD approaches that has been reported up to date. (1) The first route is thermal decomposition of precursors [using compounds like (NH_4_)_2_MoS_4_, consists of both metal and chalcogen elements] under reductive and inert environment, while the presence of H_2_ at low temperature avoids oxidation and converts (NH_4_)_2_MoS_4_ precursor directly into MoS_2_ [38]. Normally, a two-step thermolysis process was used to obtain high-quality thin film, as shown in Fig. 1a. After dip-coating of (NH_4_)_2_MoS_4_ on substrates, Ar/H_2_ mix flow were introduced and kept at relative low temperature and low pressures (500 °C, 1 Torr) for an hour to remove the residual solvent, NH_3_, H_2_S and other byproducts; then at second step of annealing, high temperature (1000 °C) and additional sulfur were applied to improve the crystallinity and electrical performance. The extra sulfur helped to remove excess oxygen and mitigate sulfur deficit caused by organic solvent evaporation [39]. Based on this, wafer scale MoS_2_ thin films were synthesized by incorporating chelating agent like ethylenediaminetetraacetic acid (EDTA) with (NH_4_)_2_MoS_4_ in DMSO solvent as a spin-coating solution, followed by heat treatment in furnace at 500–800 °C and Ar atmosphere [40]. Similarly, a series of rGO/XS_2_ heterostructures (X = W, Mo, or W and Mo alloy) were synthesized by dispersing (NH_4_)_2_WS_4_ or (NH_4_)_2_MoS_4_ into a GO solution followed by thermal treatment at low temperature [41]. (2) The second route is by sulfurization/selenization/tellurization of pre-deposited metal or metal oxide films on suitable substrate. Some techniques, such as e-beam evaporation [28], spin coating [42], atomic layer deposition (ALD) [43], and thermal evaporation [44] have been used to deposit metal or metal-containing precursors. In a typical growth, rhomboidal MoO_2_ microplates nucleated and grew on substrate through thermally evaporation, and then reduced by sulfur vapor at 650–850 °C, then MoO_2_ were sulfurized to MoS_2_ layer-by-layer at higher temperature (Fig. 1b). Because the MoO_2_ microplates were more crystalline and much larger than MoO_2_ nanoparticles, the layer-by-layer sulfurization process was much slower, which allowed to control the layer numbers by changing the high-temperature annealing time. The obtained high-quality micrometer size MoS_2_ rhomboid flakes show comparable performance with mechanically exfoliated products after being used into back-gated field effect transistors (FETs) device [45]. A continuous uniform WS_2_ films were synthesized by decorating silicon wafer through silanization reaction to improve the dispersion of precursors (WO_3_·xH_2_O), then a continuous WO_3_ formed after evaporating the solvent [46]. (3) The third route includes a simple physical vapor transport method to grow high quality monolayer or few-layers TMDs. For example, the MoS_2_ powder are involved as precursor which was kept at higher temperature zone (~ 900 °C) and finally high quality MoS_2_ was deposited on insulating substrate (placed at cooler zone, ~ 650 °C) via vapor–solid growth mechanism under Ar gas (Fig. 1c) [47]. (4) The fourth route to synthesize TMDs materials by CVD method involves vapor phase reaction of two precursor e.g., transition metal oxides/halides and chalcogen precursors. This kind of CVD process is classified into atmospheric pressure CVD (APCVD), modified metal–organic CVD (MOCVD) and low-pressure CVD (LPCVD) (Fig. 1d (i)) etc., and these are the most common techniques to synthesize TMDs layers. For CVD growth of MoS_2_ from MoO_3_ and sulfur powder, the Mo–O–S ternary phase diagram (Fig. 1d (ii)) and reaction pathways have been summarized by recent references [36]. It has been considered that MoO_3_ reacts with sulfur and produces MoS_2_ (Fig. 1d (iii)) according to the following two-step reaction,

$$2{\text{MoO}}_{3} + {\text{xS}} \to {\text{MoO}}_{{3 - {\text{x}}}} + {\text{xSO}}_{2}$$

$$2{\text{MoO}}_{{3 - {\text{x}}}} + \left( {7 - {\text{x}}} \right){\text{S}} \to {\text{xMoS}}_{2} + \left( {3 - {\text{x}}} \right){\text{SO}}_{2}.$$

But, several researchers have found that H_2_ also plays a vital role for the growth of TMDs. It acts as an additional reducing agent with Se during the growth of MoSe_2_ nanosheets [48] and thin films [49] when solid Se and MoO_3_ are used as precursors. Similarly, it has also been proven that H_2_ plays an important role for thermodynamics of the selenization of WO_3_ following the chemical reaction [50],

$${\text{WO}}_{3} + 3{\text{Se}} + {\text{H}}_{2} \to {\text{WSe}}_{2} + {\text{H}}_{2} {\text{O}} + {\text{SeO}}_{2}$$

On the other hand, due to the lower melting point of metal halides over metal sources, it is more efficient to control the ratio of metal and chalcogen. High quality single crystalline 3R-MoTe_2_ flakes were synthesized by CVD method by using MoCl_5_ as Mo precursor [51]. Very recently, molten-salt-assisted CVD method has reported for synthesizing a series of (2D) transition-metal chalcogenides (TMCs), especially for some high melting points, low vapor pressure metal and metal oxides precursors. The addition of molten salts reacted with metal precursors, and form intermediate oxychlorides which has lower melting points, thus the mass flux and reaction rate successfully is increased. This method has demonstrated to be able to synthesize 47 kinds of transition-metal chalcogenides (TMCs), including 32 binary compounds, 13 alloys, and 2 hetero-structures [52]. Similarly, some graphene-like molecules, such as reduced graphene oxide (rGO), perylene-3,4,9,10 tetracarboxylic acid tetra-potassium salt (PTAS) and perylene-3,4,9,10-tetracarboxylic dianhydride (PTCDA) can be used as growth seeds and promoted the MoS_2_ layer growth [26]. Further research have also investigated how the seeding promoters and seed concentration influence the growth of MoS_2_ monolayer [53]. It is believed that different kinds of aromatic molecules acted as a seeding promoters, deposited on various substrates and increased the surface adhesive force of MoS_2_ to improve the layer growth of MoS_2_.

### Thermodynamics and Kinetics for TMDs Growth

Three growth modes have been identified for different kinds TMDs materials including island growth (Volmer-Weber growth mechanism), layer-by-layer growth (Frank-vander Merwe growth mechanism), and mixed growth (Stranski–Krastanov growth mechanism). It is well recognized that the integration of thermodynamics and kinetics with nucleation growth provided important guidance to understand the growth mechanism as well as TMDs layers control. Hence, in this review, we mainly discuss thermodynamics and kinetics for the growth of 2D TMDs, and several mechanistic models have been introduced based on those thermodynamic and kinetic mechanisms.

**Fig. 2:**
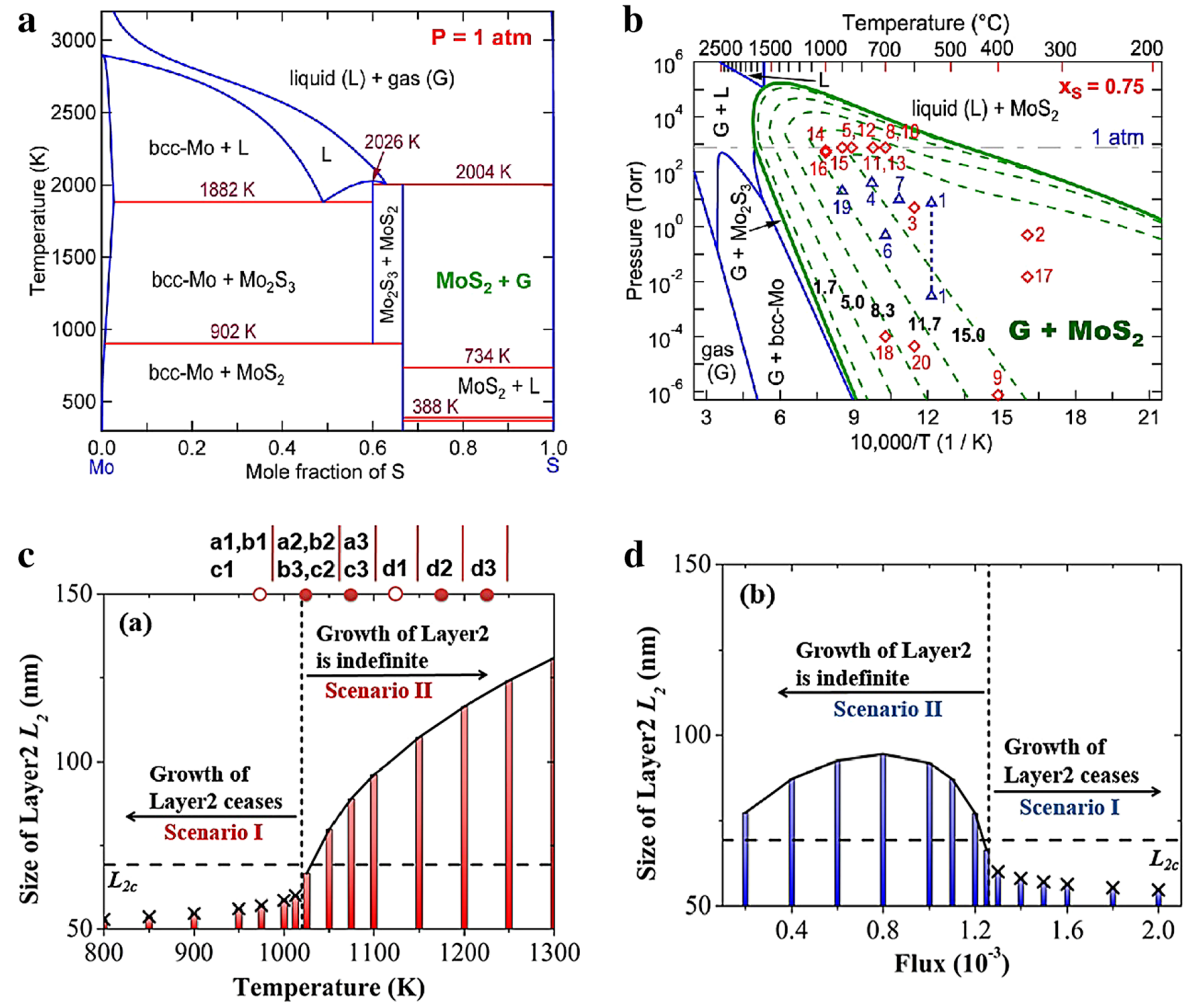
Thermodynamics and kinetics during TMDs growth. **a**, **b** CALPHAD modeled temperature–composition (T–x), and pressure–temperature (P–T) phase diagram for the Mo–S system under pressure and under the S-rich condition respectively [56]. Copyright 2016, American Chemical Society. **c** Effect of temperature and, **d** precursor flux on the growth of layer 2 for 2 s [57]. Copyright 2017, American Chemical Society

At the early stage, the thermodynamics of MoS_2_ deposition by reaction of H_2_S with molybdenum fluoride or chloride had been thoroughly studied. The chemical equilibria for Mo–S–CI–H and Mo–S–F–H system were calculated at 1 kPa as a function of temperature and reagent molar ratio $\left( \psi \right)$, H_2_S/(MoF_6_ + H_2_S) or H_2_S/(MoCl_4_ + H_2_S). The results indicated that the MoS_2_ was thermodynamically favorable to deposit in the presence of excess H_2_S [54]. Recent work proposed that partial pressure of gaseous MoS_2_ (P_Mo_) plays an important role in the control of layer number due to the thermodynamic and kinetic effects on precipitation reaction. The experimental results showed that the partial pressure must be larger than the vapor pressure (P_Mo_^o^), using the pressure difference provides the thermodynamic driving force for the precipitation reaction. So, by controlling P_Mo_, the layer number can be controlled. In particular, it is found that when P_Mo_ is between the vapor pressure of monolayer and bilayer (P_Mo,1_^o^ < P_Mo_ < P_Mo,2_^o^), exclusively monolayer were obtained. However, the larger P_Mo_ leaded to the faster precipitation [55]. According to the DFT calculated relative energies of layer-dependence of MoS_2_, and flake-size dependence of mono/bilayer MoS_2_, it has been demonstrated that the small monolayer MoS_2_ flakes were thermodynamically favorable during the initial stage of nucleation; while with the increasing of lateral size, the vertical growth become preferable. The relative energies of the semi-infinite slabs with and without graphene substrate had also been calculated in the same work and found that graphene substrate can greatly increase the critical size of monolayer flake [56]. Furthermore, the size-dependent pressure–temperature–composition (P–T–x) phase diagrams (Fig. 2a, b) were provided to predict the 2D lateral size and thickness-dependent MoS_2_ growth window. In addition, the calculated migration energy and diffusivity of Mo and S indicating that the growth of MoS_2_ is controlled by Mo due to the low diffusivity respect to Sulfur. This integrated density functional theory (DFT) and calculation of phase diagram (CALPHAD) modeling approach provided a quantitative insight into the controlled lateral and vertical growth of 2D TMDs [56].

On the other hand, the growth rate of subsequent layer depends on the sizes of initial layer and subsequent layer. Recently, a multiscale model has been developed and found that a maximum size of layer-1, while minimum size of layer-2 ensured vertical growth according to thermodynamic criterion. The increasing size of layer-1 decreased the growth rate of layer-2, while the size of layer-1 reaching the maximum size, the growth of layer-2 ceased to grow. A successful vertical growth happens when the size of layer-2 exceeds the critical size before layer-1 reaching maximum size. By changing the kinetic parameters (growth temperature and flux), it was demonstrated that the monolayer was obtained at relatively low temperature but high gas flow rate conditions (Fig. 2c, d), consistent with the trend of growth of bilayer graphene at decreased supply of adatoms [57, 58]. In order to investigate and predict vdW epitaxy growth process to guide experimental study, a Kinetic Monte Carlo (KMC) simulation method was used to investigate the complex competitions in growth rate, morphology, homogeneous nucleation and layer number. Extrinsic parameters (including temperature, adsorption rate, chalcogen to metal ratio of the precursors), intrinsic parameters, site energy, adsorption energy and transition energy barriers, as well as substrate effects were introduced to provide a fundamental understanding about the growth mechanisms at atomic scale [59]. Although this model was set for molecular beam epitaxy (MBE) growth of TMDs, after further refinements it applied to predict CVD growth qualitatively and quantitatively.

## Controllable growth for TMDs materials

The potential applications in electronic and optoelectronic devices has demanded the scalable synthesis of uniform, high-quality TMDs layers. Generally, high-quality refers to high crystallinity, large domain size, defect-free, and limited grain boundaries. The quality of large scale uniform MoS_2_ layers largely depends on property factors such as grain size and boundaries, orientation and their morphology, as discussed below.

### Domain size and grain boundaries

**Fig. 3:**
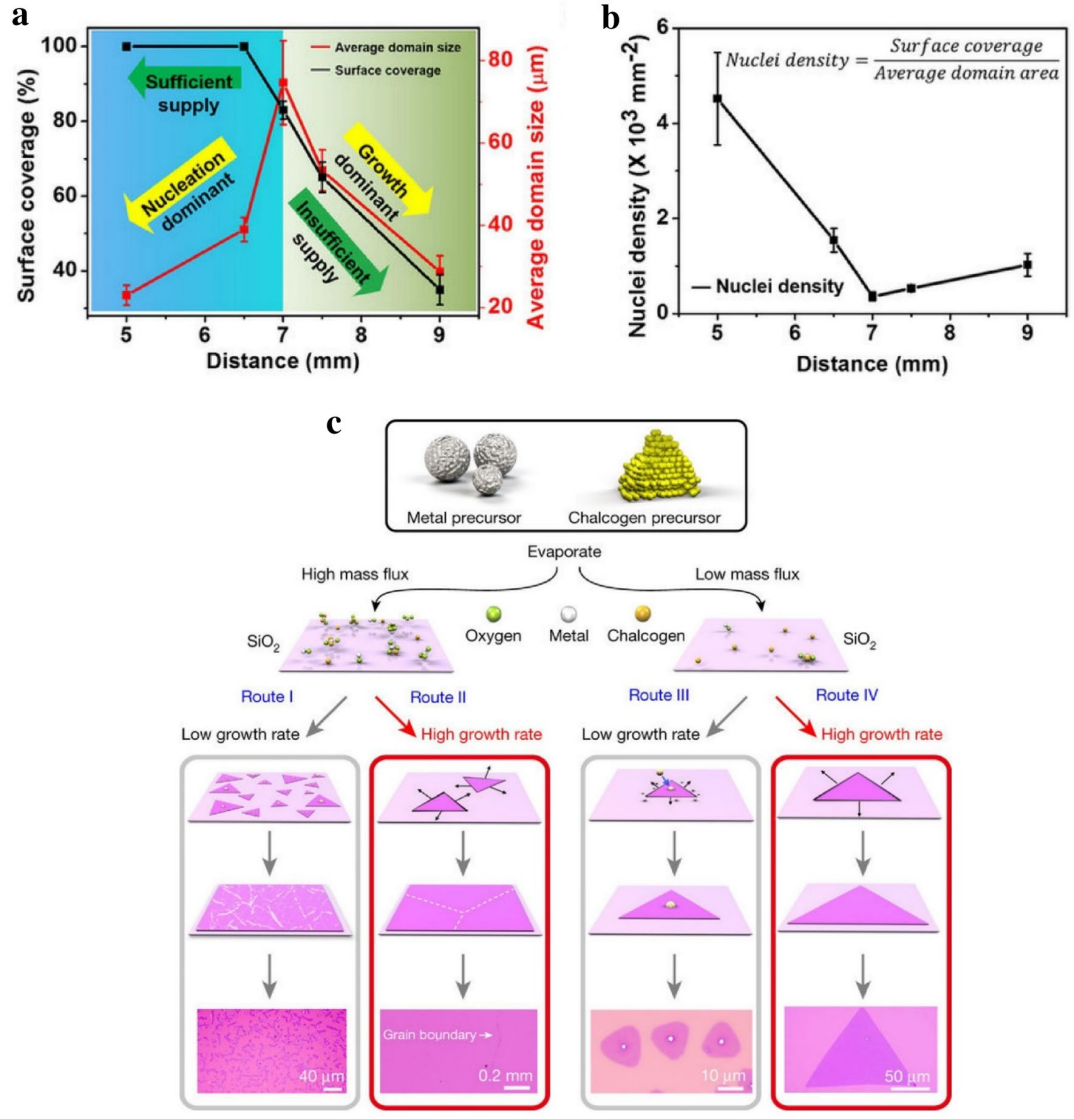
Grain size and boundaries control. **a** Surface coverage and average domain size of MoS_2_ as a function of precursor/substrate distance, **b** effect of precursor/substrate distance on the nuclei density of MoS_2_ during growth [62], Copyright 2015, Springer Nature. **c** Flow diagram represents different routes for the synthesis of distinct types TMCs by the chemical vapor deposition method. Different routes indicated the nucleus formation and their growth mechanism as a function of mass flux of precursor and growth rate [52]. Copyright 2018, Springer Nature

Previous report has shown that large-scale polycrystalline MoS_2_ film with a tunable grain size up to micrometers can be synthesized via a new CVD configuration. When the grain size increased from 20 to 600 nm, Raman spectra showed nearly no difference in peak position and intensity, but obvious PL shift was observed due to band-gap modulation. Moreover, because of the extra scattering at grain boundaries, it was clear that the carrier mobility was impaired when grain size reduced [60]. Furthermore, recent research showed that the low angle grain boundaries of CVD-grown polycrystalline MoS_2_ deteriorate thermal conductivity [61]. It has widely acknowledged that the nucleation density is closely related to the grain size. The following equation shows the relationship between source and growth distance (*d*) with thermodynamic and kinetic growth factor—the concentration of gaseous MoS_2_ (*Cg*):

$$C_{g} \left( {d,t} \right) = C_{g} \left( {0,t} \right)exp\left( { - \frac{{d^{2} }}{4Dt}} \right)$$

where *t* is the time, and *D* is the diffusion constant. Figure 3a summarized the surface coverage and the average domain size as a function of distance. When the distance was less than 7 mm, high *Cg* ensured sufficient source supply and high nuclei density results many small MoS_2_ domains adjacent, coalescent, and overlap at boundary with high surface coverage. While the largest single-crystal domain size (> 300 μm) was obtained at sufficient source supply but suppressed nuclei density condition (*d* = 7 mm) (Fig. 3b). However, because of the low *Cg* at higher distance (*d* > 7 mm), both average domain size and surface coverage decreased due to the insufficient source supply [62].

Depending on various mass flux and growth rate, the growth of 2D TMCs is divided into four routes (Fig. 3c). The formation of nucleus and the growth of domains depends on mass flux, especially metal precursor, while the grain size is determined by growth rate. High mass flux of metal precursor but low growth rate tends to form polycrystalline film with small grains and lots of grain boundaries (Route I), while high growth rate can produce smoother monolayer film with large grain size and limited grain boundaries (Route II). However, at low mass flux, it prefers to form single-crystalline with small domains at low growth rate (Route III), large monolayer single-crystal at high growth rate (Route IV) [52]. In order to decrease the nucleation density, without changing the amounts of precursors or growth parameters, some additional attempts have been successfully made. For example, NiO foam was used to grow centimeter scale continuous monolayer MoS_2_ by trapping chemical precursors [63]. Metal precursors (MoO_3_) reacted with NiO and form a Ni–Mo complex, thus the precursor concentration as well as nucleation density decreased. Recently, “liquid-state” substrates show a new possibility to grow large grain size TMDs materials. Because of the low nucleation density together with low migration coefficient, large MoSe_2_ and MoS_2_ monolayer single-crystals up to ~ 2.5 mm successfully deposited on the low-defect and homogeneous molten glass surface [64]. Similarly, another report has further demonstrated that the uniform distribution of Na in soda-lime glass will promote the growth rate of monolayer MoS_2_. A face-to-face metal precursor supply route was designed to synthesize 6-inch uniform monolayer MoS_2_ with grain edge length larger than 400 μm. The DFT calculations proved that during the MoS_2_ growth, the energy barrier was reduced with Na adsorption [65]. On the other way, due to the different facet-dependent binding energy between Au and MoS_2_, it is found that at relative high growth temperatures, large single-crystal domains prefer to grow on Au (100) and Au (110) than on Au (111) facets [66].

The grain boundary is another important factor that significantly affects the quality of layered TMDs, and therefore it is essential to understand the grain boundary formation mechanism, as well as the electric and optical performance at boundary. Two possible modes are observed for MoS_2_ boundary formation during vapor phase growth process. The traditional grain boundaries growth involves formation of chemical bonds between two single layer grains, where in-plane growth is stopped but the boundary site contributes to the nucleation of second layer. While the other mode of boundary formation involves no chemical bonds, with two grains overlap and continue to grow on top of each other [67]. At the same time, the optical and electronic properties of faceted tilt and mirror twin grain boundaries in poly-crystalline MoS_2_ has been investigated, observing strong enhancement of PL and slight decrease of electrical conductivity at faceted tilt boundaries, with the opposite at the mirror twin boundaries [68]. This has been further demonstrated that the PL mapping can be used to identify grain boundaries in MoS_2_ quickly due to the thermal mismatch induced non-uniform tensile strain [69]. Meanwhile, it is also found that 60° grain boundaries, either point sharing or edge sharing, both consists of distinct fourfold ring chains and exhibit metallic behavior. Further work has provided insight into various small-angle (18.5°, 17.5°) grain boundaries possessing distinct kinds of dislocation core structures. The interaction of grain boundaries with point defects showed the possibility to control the precise grain boundary [70].

### Orientation

As mentioned above, the grain boundary formed from different misorientation of adjacent grains, which leads to distinct electronic structures across the boundary [71]. In most cases, the distorted grain boundaries with high misorientation angles and defects lowered the quality of TMDs as electronic devices. Therefore, like grain-size controlled strategy, control the grains orientation as well as highly aligned growth of 2D TMDs are also important to promote the formation of large-size uniform layers. Two mechanisms have been developed to explain the dominant 60° misorientation. In the first scenario, the small 2D islands were pre-aligned at 60°; while at the second scenario, the random orientations of islands were drawn by capillary forces into 60° [72].

**Fig. 4:**
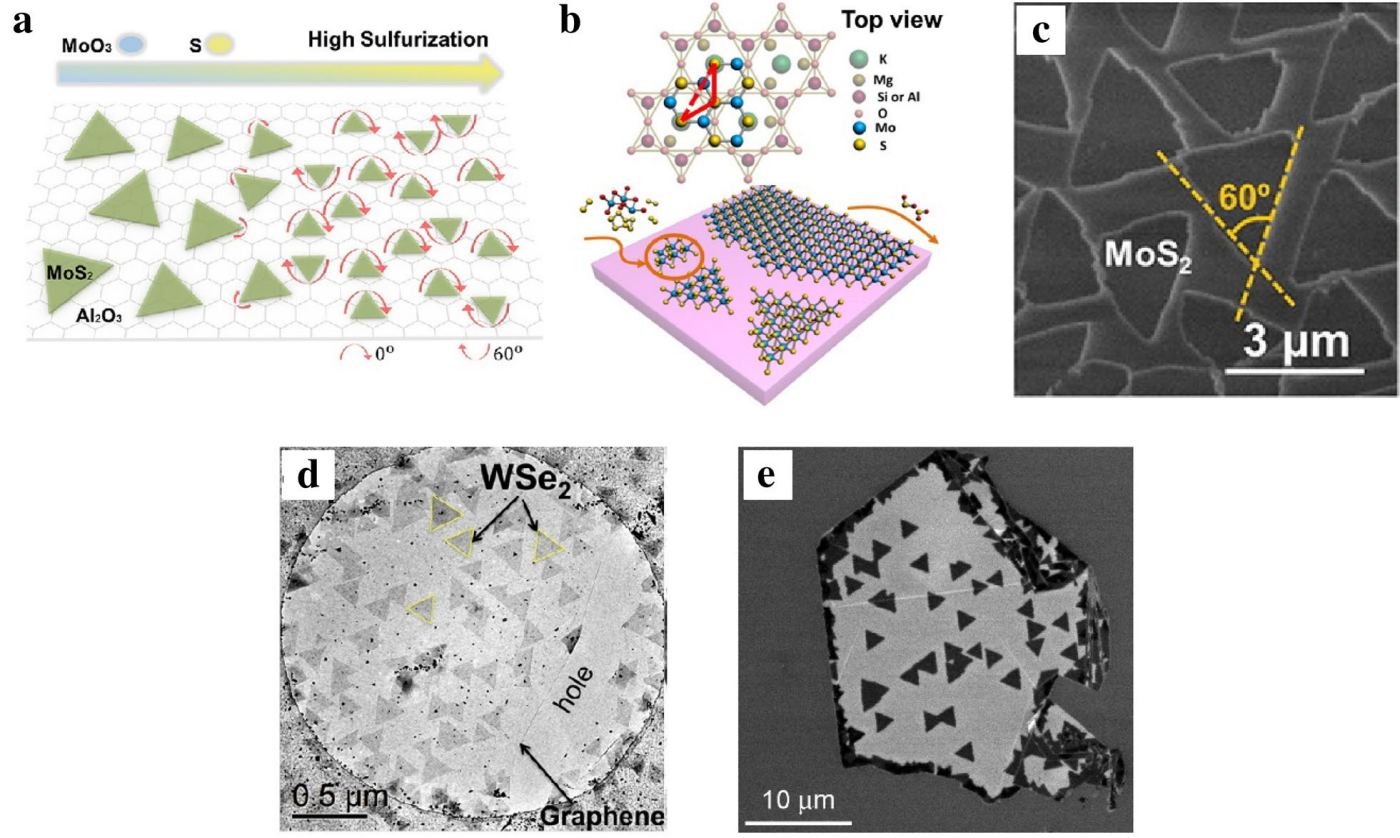
Orientation control of TMDs materials. **a** Rotation of MoS_2_ seeds as a function of precursor ratio (S/MoO_3_) during initial nucleation steps [75]. Copyright 2017, American Chemical Society. **b** Epitaxial growth of MoS_2_ on mica surface. **c** SEM image of MoS_2_ on mica [76]. Copyright 2013, American Chemical Society. **d** WSe_2_ on the freestanding graphene membrane [78]. Copyright 2015, American Chemical Society. **e** SEM image of WS_2_ on h-BN flake [80]. Copyright 2014, American Chemical Society

Recently, many aligned growths have been successfully achieved on single crystal substrates such as c-plane (0010) facet sapphire, mica, or GaN [73], which guided about TMDs growth orientation by step-edges at relative high temperature, or through van der Waals (vdW) interaction due to the lattice match between substrate and TMDs [74]. Moreover, it has been demonstrated that the small MoS_2_ seeds easily rotated to favorable orientations (0° or 60°), due to the low potential energy between MoS_2_ seed and c-plane sapphire substrate. While the ratio of precursors (S/MoO_3_) plays a key role to form a rotatable seed at initial nucleation step (Fig. 4a) [75]. Similarly, mica substrate was used for such synthesis, shown in Fig. 4b, c and it is reported that the nearly lattice-matching property result at a relative lower growth temperature (~ 530 °C), and the triangle MoS_2_ seeds were primarily aligned along two opposite orientations [62, 76]. Further work revealed that high concentration of hydrogen helped to form an active Al-rich sapphire surface, enhancing the interaction between WS_2_ and sapphire, thus formed well oriented triangle and effective stitch of merged grains [77]. Apart from single crystal substrates, the underlying high-symmetry materials, such as graphene or hexagonal boron nitride (h-BN) also used to align the triangle domains at 60°. Recent literatures have shown that WSe_2_ triangles were orientated as similar patterns on graphene, results epitaxial growth of monolayer WSe_2_ (Fig. 4d). On the other hand, selected area electron diffraction (SAED) results showed misorientation of MoS_2_ on graphene producing non-epitaxial monolayer growth [78]. Although MoS_2_ grains showed random orientations on graphene according to previous results, at the same time, other work showed that sides of single-crystalline MoS_2_ preferred parallel orientation at one sides of underlying graphene, and the SAED result also confirmed the lattice orientations of MoS_2_ and graphene coincided [79]. Similarly, another group observed distinct crystallographic orientation of WS_2_ on h-BN with two different orientations with 60° angle, shown in Fig. 4e. Unlike graphene, the difference in electronegativity between B and N atoms results coulombic interaction played a vital role for the limited crystallographic orientation of WS_2_ on h-BN substrate [80].

### Morphology

**Fig. 5:**
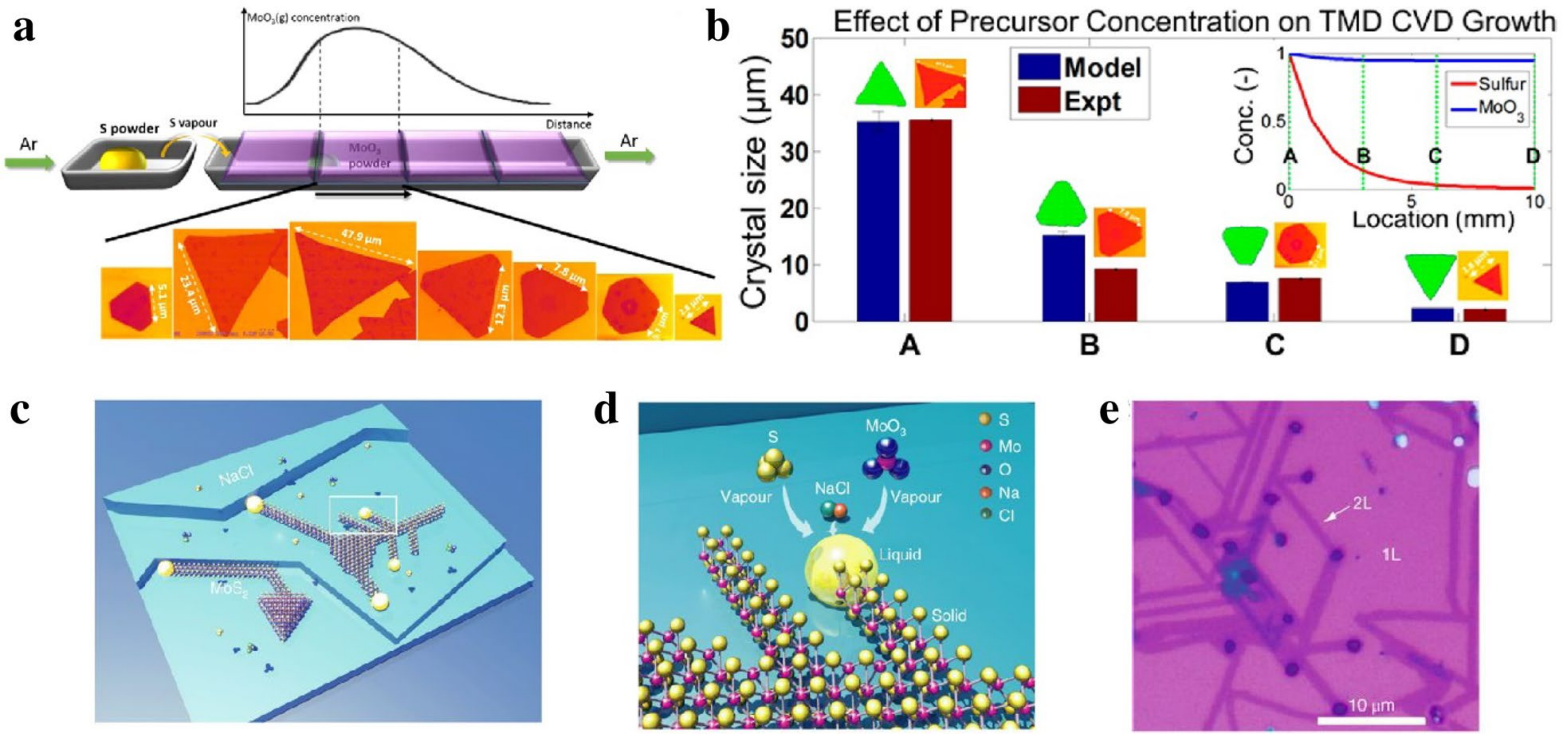
Morphology control of TMDs materials. **a** MoS_2_ CVD growth system. The optical images show the MoS_2_ with various sizes and shapes [84]. Copyright 2014, American Chemical Society. **b** Effects of precursors concentration on the sizes and shapes of TMDs during CVD growth measured by both theoretically and experimentally [85]. Copyright 2016, American Chemical Society. **c**, **d** MoS_2_ ribbons grow on NaCl crystal surface via VLS mode. **e** Optical image of MoS_2_ ribbons on a monolayer MoS_2_ film [87]. Copyright 2018, Springer Nature

**Fig. 6:**
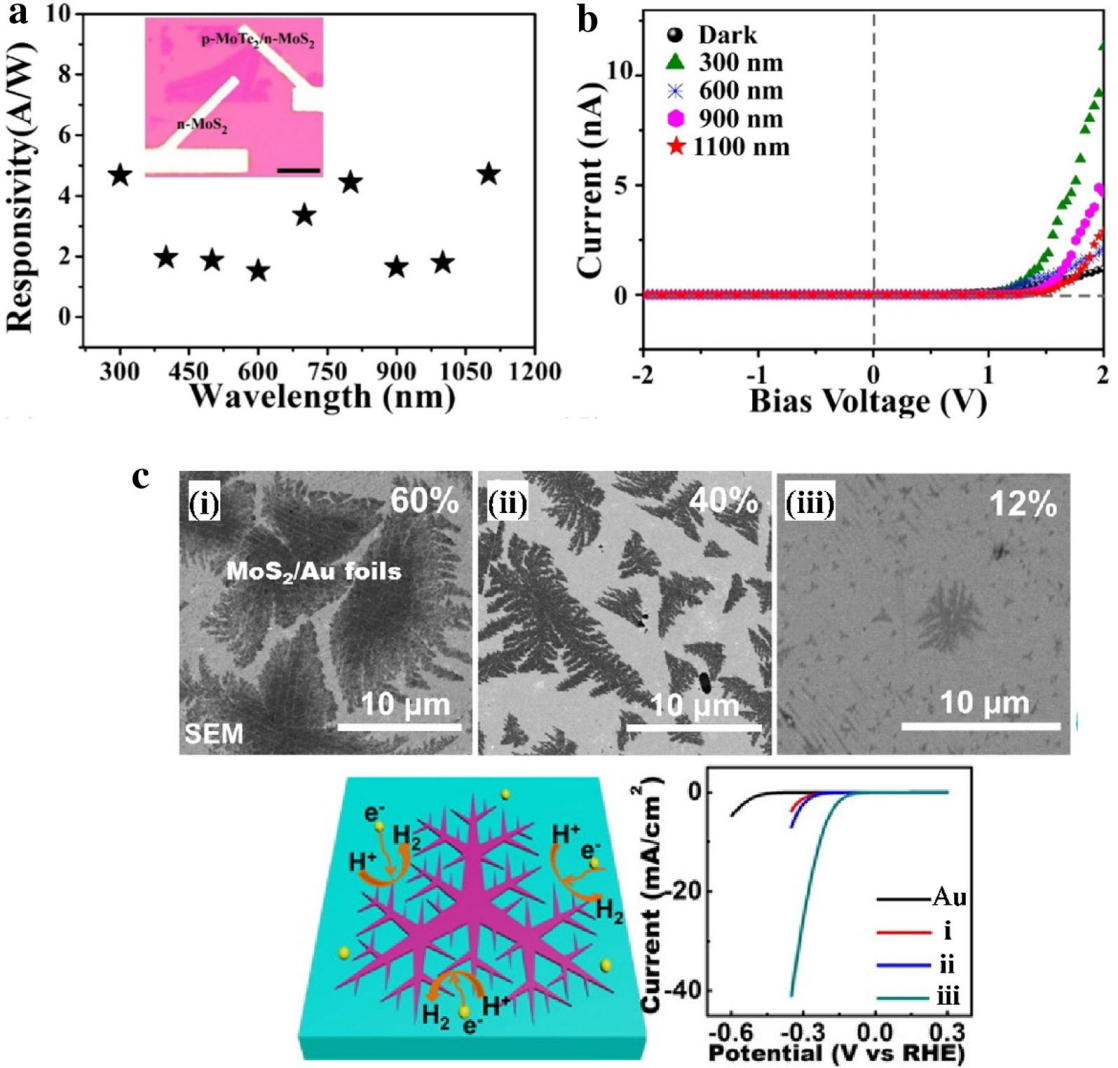
Optoelectronic properties and electrochemical catalysis applications of TMDs materials. **a** Spectral responsivity curve of the MoTe_2_/MoS_2_ heterostructures **b** I–V curves of the phototransistor device [89]. Copyright 2018, Elsevier B.V. **c** SEM images of different coverages of dendritic MoS_2_ and their polarization curves [95]. Copyright 2014, American Chemical Society

The morphology control is also important for scalable synthesis of high quality TMDs, which further ensures high quality and high performance for applications in optoelectronic and electronic properties. Single-layered TMDs with different morphologies, like triangles, stars, pentagons, hexagons, etc. have been synthesized by CVD methods [81]. Many researchers demonstrated that the morphologies of TMDs layers are dependent on the growth conditions, including substrates, gas flow rate, temperature, precursors ratio and concentration, etc. For example, it is found that the hydrogen has substantial effect on the morphologies of TMDs during growth, and the shape of monolayer triangle WS_2_ can be tailored from jagged to straight edge by adding minor amount H_2_ gas [82]. Similarly, with increasing H_2_ concentration, more WO_3_ were reduced by H_2_ to form volatile WO_3−x_, by depositing W atoms onto Se edges. While appropriate fluxes of WO_3−x_ and Se enables the formation of triangles with W/Se edges or hexagon with high selectivity by controlling the H_2_ pressure [83]. Moreover, the distance between metal source and substrate, which determines the concentration of vapor metal precursor, plays a crucial role in the MoS_2_ morphology control [84]. With the increasing of distance from MoO_3_ precursor, MoS_2_ is transformed from mid-sized truncated triangle of ~ 6 μm to large triangle of ~ 50 μm, then increase to medium-sized truncated triangle, followed by small hexagon, and finally tiny triangle of ~ 2–3 μm (Fig. 5a). This shape evolution parameters was found to strongly depends on the growth rates of different edge terminations. When the Mo: S ratio was equal to 1:2, the growth rate of different edge terminations was equal, which resulted hexagon shape. On the other hand, when Mo: S ratio was larger than 1:2, the S-zigzag (zz) terminations grew faster than the Mo-zz terminations because the exposed S atoms had higher probability to bond with free Mo atoms, thus the final structure turned to triangles with Mo-zz termination sides. Conversely, when the substrate was far away from Mo source, the concentration of Mo vapor was decreased while the S vapor gradient was small, then the Mo: S ratio was smaller than 1:2, and S-zz triangles were obtained. Meanwhile, some work proposed that the Mo-zz triangle has sharp and straight edges than S-zz triangle, which allowed the identification of crystal orientation by optical microscopy [68]. Furthermore, a growth model is developed using KMC simulations to predict the MoS_2_ variation in shape and size as a function of precursor concentration (Fig. 5b) [85]. In addition, a combined experimental and numerical simulation work is used to control the synthesis of vertical to planar 2D MoS_2_. According to the result, the transition from vertical to planar triangle and thin film growth was achieved by placing substrate at different position with different orientations and changing the carrier gas flow rate. When the substrate was normal to the flow stream, the vertically standing MoS_2_ nanosheets were the dominant structures; while planar triangle and thin film were synthesized by placing the substrate facing down on top of the crucible and facing up next to metal source. The concentration gradient and distribution of the precursor play the critical role to control the growth mode and density, resulting in different orientations and morphologies [86]. Very recently, a vapor–liquid-solid (VLS) mode has been promoted to grow monolayer MoS_2_ nanoribbons. The liquid phase Na–Mo–O droplet formed by alkali metal halide reacting with metal oxide precursors that worked as intermediate and crawl on the substrate (Fig. 5c, d) [87]. Using this method, locally well-defined MoS_2_ ribbons have been further grown on a continuous MoS_2_ monolayer film, which pre-grown on a SiO_2_/Si substrate (Fig. 5e). To meet the practical demands, lots of attempts have been made to synthesize large-scale continuous 2D binary films or lateral heterostructures for electronics, optoelectronics, sensors, or other flexible devices. For example, a two-step CVD method is reported for epitaxial growth of lateral WSe_2_–MoS_2_ heterojunction with an atomically sharp interface. This kinds of TMDs lateral heterojunctions are key components for p-n rectifying diodes, light-emitting diodes (LED), photodetectors, and photovoltaic devices, etc. [88]. Recently, 2H-MoTe_2_/MoS_2_ were synthesized by using a magnet-assisted precursor delivery system. Based on this heterostructure, the vertical p–n junction shows a broadband photo-response range from UV (200 nm) to near-infrared (1100 nm) (Fig. 6a) [89]. Figure 6b illustrates the MoTe_2_/MoS_2_ device shows a photovoltaic p–n junction effect under bias voltage for incidental lights, compared with dark environment. Other work also demonstrated that MoS_2_–MoS_2_/CNT heterostructure exhibits attractive electrical and mechanical properties for flexible optoelectronics [90]. However, for electrochemical catalysis application and energy storage, like hydrogen evolution reaction (HER) and battery materials, it has been proven that the active sites are mainly at the edges or defects of MoS_2_. The exchange current density shows a linear dependence on the MoS_2_ edge, rather than area coverage [91, 92]. Only the surface of few TMDs martials from group V (such as V, Nb, and Ta) showed activity for electrolysis of water [93]. Besides these, sufficient amounts of efforts have been drawn to grow vertically aligned or dendritic TMDs to increase active site for HER. For example, synthesized MoS_2_ and MoSe_2_ film with vertically aligned layers are compared and confirmed the exchange current density has direct correlation with the density of the exposed edge sites [28]. Similarly, horizontally and vertically aligned MoS_2_ by CVD method has shown that the vertically aligned MoS_2_ with a hydrophobic surface exhibit better HER performance [94]. On the other hand, much attention has been paid on the substrate effect for synthesizing dendritic MoS_2_ on specific substrates. Dendritic, monolayer MoS_2_ flakes on SrTiO_3_ (STO) is obtained and the tunable degrees of fractal shape provide abundant edges and serve as effective electrocatalysts for HER (Fig. 6c) [95]. After that, it is also reported that dendritic MoS_2_ on LaAlO_3_(100) is also engineered to expose more active edge sites with high nucleation density [96]. Our group has also demonstrate the growth of single-crystal MoSe_2_ on N-doped graphene where polymer worked as an absorption matrix to mitigate aggregation, and the H_2_ etching step during CVD treatment provided extra edge sites for H_2_ evolution in the inert basal plane [97].

Recently, the evolution of novel structures of TMCs, like polygonal twins, screw dislocations, etc. is also widely studied because the possibility of new functions. The merging of single-crystal or changes of layer stacking not only form different morphologies, but also affect the optical, electronic, and magnetic properties of TMCs. Recent report has shown that the preparation of a spirals of layered MoS_2_ with AA lattice stacking morphology (contrast to centrosymmetric AB stacking in intrinsic MoS_2_), this non-centrosymmetric spiral leads to second-harmonic generation (SHG) signal [98]. It is further confirmed that the triangular spiral WSe_2_ plates with strong SHG and enhanced PL signal, and the plates contain both triangular and hexagonal dislocations with diverse mixed properties [99]. Wulff constructions is used for bowtie- and star-shape MoS_2_ twin-islands (high-symmetry poly-crystals with 60° lattice misorientation angles) under thermodynamic and or kinetic control conditions. Based on the comparison of the length of grain boundary and base edge, it is possible to distinguish the thermodynamic and kinetic control by measuring the aspect ratio of bowtie shape. The phase field model was also employed to simulate the growth of MoS_2_ and symmetric shape evolution [72]. Successfully growth of dendrites MoS_2_ is demonstrated by pretreating substrates with adhesive tapes and controlling S:Mo vapor ratio. The successive nucleation of twin crystals at the side edges contributed to the sub-branch growth, and the accumulated sulfur vacancies in cyclic twin regions lead to strong and localized enhancement of PL emission, determined the shape dependency of optical property [100].

## Summary and outlook

Recent progress of 2D TMDs materials brings lots of advances and opportunities in the scientific community with numerous concepts and technologies. There have been many breakthroughs in the synthesis of TMDs layers by CVD techniques in the past several years. In this review, we analyze the possibility of controllable growth of 2D TMDs by CVD method based on the discussion of growth routes and mechanisms. The control of grain size, boundaries, orientation and morphology are highlighted, aiming to bring inspiration to future research in this field.

Although the latest published researches have successfully achieved the growth of high quality inch-size TMDs, it is still challenging to realize the controllable growth of wide range of TMDs with desired layer number, large domain size, target orientation and morphologies. One of the most important reason is that most of the precursors of TMDs are solid-state in common CVD growth system, except MOCVD. Unlike gas sources for graphene, it is difficult to control and maintain the concentrations of precursors precisely over whole growth process. Additionally, by using inductively coupled plasma enhanced CVD, graphene can be synthesized at temperature as low as 300 °C, but it is still challenging to grow TMDs at such low temperature. These show the importance of development of new CVD system, such as using external heating equipment, or introducing additives to lower melting points and reaction barriers. Meanwhile, suitable analytical model combined with simulations and calculations should be further developed to reveal the growth mechanism and set reliable theoretical predictions to control the growth experimentally.

On the other hand, 2D TMDs layers are promising materials for different kinds of electronics and optoelectronics devices. Although most of the TMDs based on VIB group metal, like MoS_2_, WS_2_, MoSe_2_ and WSe_2_ have widely been synthesized by CVD method, the growth of other novel layered materials may open up the possibility to explore the applications of 2D materials. Furthermore, the defects engineering, grain boundaries and phase transitions of TMDs display tremendous opportunity for specific area of applications. Additionally, we mainly focused on the synthesis of binary compounds in this review, but it is worth noting that the alloying of semiconductors and construction of heterostructures are ideal ways to tailoring the band structure of TMDs. Hence, the design and preparation of novel TMDs heterostructures as well as alloys is also important aspects to create new practical applications. Some other issues, such as the direct growth on flexible substrates or a successful method to transfer the TMDs on the target substrate after growth is necessary for the fabrication of most electronic devices. Therefore, considering the growth and application of 2D TMDs layers are still at the exploration stage, there are still numerous possibilities and strong demand to further understanding and developing the synthesis of 2D TMDs materials.
